# Evaluation of Reliability of Ultrasonographic Parameters in Differentiating Benign and Metastatic Cervical Group of Lymph Nodes

**DOI:** 10.1155/2014/238740

**Published:** 2014-04-17

**Authors:** Chintamaneni Raja Lakshmi, M. Sudhakara Rao, A. Ravikiran, Sivan Sathish, Sujana Mulk Bhavana

**Affiliations:** ^1^Department of Oral Medicine and Radiology, Drs. Sudha and Nageswara Rao Siddhartha Institute of Dental Sciences, Gannavaram Mandal, Krishna District, Andhra Pradesh 521286, India; ^2^Department of Otorhinolaryngology, Dr. Pinnamaneni Siddhartha Institute of Medical Sciences and Research Foundation, Gannavaram Mandal, Andhra Pradesh 521286, India; ^3^Department of Oral Medicine and Radiology, Sibar Institute of Dental Sciences, Takkellapadu, Guntur, Andhra Pradesh 522509, India; ^4^Department of Oral Medicine and Radiology, Chettinad Dental College & Research Institute, Rajiv Gandhi Salai, Kelambakkam, Kanchipuram District 603103, India

## Abstract

The aim of the current study is to determine the efficacy of ultrasound in differentiating between benign and metastatic group of cervical lymph nodes. The study included forty-five subjects who were divided into three groups with 15 in each, by stratified random sampling method. Group 1 comprised fifteen patients without signs and symptoms of any infection and neoplasms in head and neck region (control group). Group 2 included fifteen patients with signs and symptoms of malignancy in head and neck region. Group 3 consisted of fifteen patients with signs and symptoms of odontogenic infections. “MY LAB-40” ultrasound machine with linear array transducer of 7.5 MHZ frequency was used for detecting cervical lymph nodes following Hajek's classification. The patients further underwent ultrasound guided FNAC under standard aseptic protocol and samples were subjected to cytopathological evaluation. Chi square analysis and one way ANOVA test were applied to obtain the results. We concluded that ultrasound and USG FNAC can be used accurately to assess the status of lymph nodes. The ultrasonographic features of lymph nodes with round shape, absence of hilar echo, sharp nodal borders, hyperechoic internal echogenicity, and presence of intranodal necrosis were highly suggestive of metastatic cervical lymph nodes.

## 1. Introduction

Imaging techniques play a very important role in diagnosing head and neck pathologies especially those involving deeper soft tissues. Lymphadenopathy is one such condition where critical evaluation becomes mandatory not only to assess the severity of the disease but also to determine disease prognosis and proper treatment planning. Clinical examination of cervical lymph nodes is important in such patients but mostly remains difficult owing to their diverse location and multiple numbers. Ultrasound has higher sensitivity (96.8%) than palpation (73.3%) for detection of cervical lymph nodes [1]. CT and MRI can be used for evaluation of lymph nodes, but they are less sensitive than ultrasound in detecting nodes <5 mm in diameter [2], whereas ultrasound can detect nodes even less than 2 mm in diameter [3]. Ultrasonography has gained recent popularity in maxillofacial imaging as it is nonionizing, noninvasive, and cost effective [4, 5].

Fine needle aspiration cytology is rapid, safe, simple, and nonexpensive diagnostic technique. Ultrasound guided fine needle aspiration cytology showed improved diagnostic accuracy with 97.9% sensitivity and 100% specificity more than conventional fine needle aspiration cytology [6, 7].

Hence the study was designed to evaluate the reliability of grey scale ultrasound in differentiating the pathologies of cervical lymph nodes and to emphasize its sensitivity and specificity.

## 2. Materials and Methods

The study included forty-five subjects who were divided into three groups by stratified random sampling method.

The inclusion criteria for each group were as follows. Group 1 comprised fifteen study samples without signs and symptoms of infection and neoplasms in head and neck region. These were considered as control group. Group 2 included fifteen patients with signs and symptoms of malignancy in head and neck region such as persistent ulcers or proliferative overgrowths, unexplained tooth mobility not associated with periodontal disease, all red or red and white lesions on the oral mucosa, hoarseness of voice, persisting dysphagia, and unresolved neck masses. Group 3 consisted of fifteen patients with signs and symptoms of infections in head and neck region such as odontogenic infections in dentoalveolar region.

Exclusion criteria were patients with granulomatous infections like tuberculosis, sarcoidosis, and so forth; HIV associated lymphadenopathy; nonspecific lymphadenopathy; benign lymphadenopathy conditions like Kikuchi's disease, Kimura's disease, Rosai-Dorfman disease; and primary lymph node malignancies like lymphomas. The study was conducted after obtaining clearance from the institutional ethical committee.

**Figure 1:**
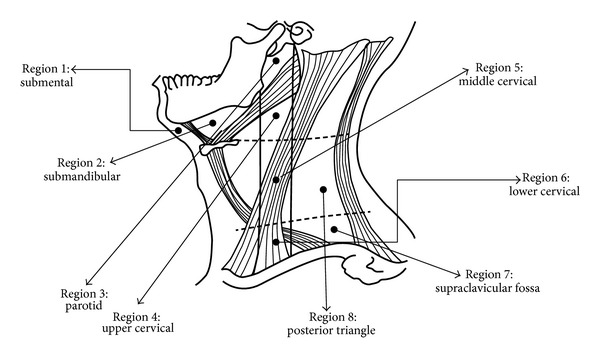
Hajek's classification for ultrasound examination of cervical lymph nodes.

The patients satisfying all the inclusion and exclusion criteria were informed about the study in detail and consent was obtained. All the procedures were in accordance with Declaration of Helsinki. Patients willing to be a part of the study were subjected to thorough clinical examination. In all group 2 individuals primary site of malignancy was confirmed by incisional biopsy. Ultrasound scanning was then performed by a single experienced radiologist who was blinded regarding the clinical diagnosis. “MY LAB-40” ultrasound machine (ESOATA Biomedica Ltd.) with linear array transducer of 7.5 MHZ frequency [8, 9] was used to study detectable cervical lymph nodes following Hajek's classification (Figure 1) [10]. Grey scale sonographic features considered for analysis of cervical lymphadenopathy were as follows:

- size of the lymph node: assessed by measuring maximal transverse diameter;
- shape of the lymph node: assessed considering the ratio of short axis to long axis (S/L); if S/L ratio was less than 0.6 they were considered as round shaped nodes and if it was more than 0.6 they were considered as oval shaped nodes [11];
- nodal borders: classified as sharp or smooth;
- internal echogenicity: classified as hypo- or hyperechoic;
- echogenic hilum;
- nodal necrosis were assessed and recorded whether present or absent.

All the above mentioned ultrasound criteria were assessed and recorded in patients proforma.

The patients further underwent ultrasound guided FNAC under standard aseptic protocol and samples were subjected to cytopathological evaluation and were graded as follows:

 group 1: positive—evidence of pathology (reactive inflammatory cell),
 negative—no evident pathology/normal study;
 group 2: positive—with evident dysplastic features,
 negative—no evident pathology/normal study or evidence of reactive inflammatory cells;
 group 3: positive—with evident reactive inflammatory cells,
 negative—no evidence of any pathology/normal study or evident dysplastic features.

The cytopathological diagnosis was considered as gold standard. The obtained data from ultrasound examination and FNAC were tabulated for correlation and statistical analysis.

## 3. Results

The obtained data was subjected to Chi square analysis and one way ANOVA test. The demographic data were depicted in Figures 5 and 6. Mean size of lymph nodes in group 1 was 0.82 mm, in group 2 was 2.29 mm, and in group 3 was 1.24 mm with highly significant *P* value of 0.0000 when compared with one way ANOVA test as shown in Tables 1 and 2. Percentage distribution of study subjects according to ultrasound criteria like shape, nodal borders, echogenic hilum, internal echogenicity, and intranodal necrosis for differentiation of benign and metastatic cervical group of lymph nodes, along with obtained *P* values, was illustrated in Figures 7, 8, 9, 10, and 11 which revealed statistically highly significant values (*P* < 0.05). On USGFNAC, group 1 samples showed 20% positive results and 80% were negative; group 2 revealed 100% positive results. Group 3 showed 86.67% positive results and 13% negative results as represented in Figure 12. Sensitivity and specificity of ultrasonographic criteria for differentiation of benign and metastatic group of cervical lymph nodes were depicted in Table 3.

## 4. Discussion

Thorough clinical evaluation of cervical lymph nodes will be a difficult task as there are about 300 cervical lymph nodes in the neck varying in size from 3 to 25 mm which were embedded within soft tissues of the neck. Especially in head and neck malignancies presence of metastatic nodes on one side of the neck reduces 5-year survival rate to 50% where as bilateral involvement of neck further reduces survival rate to 25%. Henceforth cervical lymphadenopathy assessment is vital as it aids in selection of treatment modalities and predicting prognosis [11, 12]. Metastatic cervical lymph nodes are site-specific. In patients with a known primary tumour, the distribution of metastatic nodes assists in tumour staging; however, if the primary tumour is not identified, the distribution of proven metastatic nodes may give a clue to identify the primary tumour [13, 14].

The present study showed that the age and gender distribution in group 1 (control group) and group 3 was nearly the same in the patients being in second decade, whereas mild male predilection in group 1 and female predominance in group 3 were noted. The metastatic group had mean age of 57.87 with male predominance of 66.67%.

Ultrasound was used to assess the normal, metastatic, and reactive lymph nodes in the present study by observing certain sonographic features like size, shape, nodal borders, echogenic hilum, internal echogenicity, and intranodal necrosis.

In the current study the mean average size of normal cervical lymph nodes was 0.82 cm, metastatic cervical lymph nodes was 2.29 cm, and reactive cervical lymph nodes was 1.24 cm with highly significant *P* value (*P* = 0.0000). These results were in accordance with Hajek et al. and Solbiati et al. The upper limit of the maximal short axis axial diameter for normal cervical nodes is controversial with two values being considered: 5 and 8 mm [15, 16]. However, Bruneton et al. reported that normal cervical lymph nodes had a maximal short axis axial diameter of 8 mm or less [17]. Generally malignant nodes tend to be larger; however inflammatory nodes can be as large as malignant nodes and in contrast metastatic deposit can be found in small nodes [10].

Most investigators have suggested short axis/long axis ratio as the most reliable indicator for metastatic nodes [18, 19]. In this study 86.6% of metastatic cervical lymph nodes were round in shape (short axis/long axis ratio > 0.60) when compared to normal and reactive nodes which were oval (short axis/long axis ratio < 0.60) with significant *P* value (*P* = 0.0015) as observed by Toriyabe et al. where 68% of benign nodes S/L ratio was less than 0.6 and 81% of metastatic nodes ratio was more than 0.6 and round in shape [11]. Yusha et al. reported that short axis/long axis diameter ratio >0.73 (round) indicates metastatic node when compared to the reactive cervical lymph nodes with ratio <0.54 [20].

**Figure 2:**
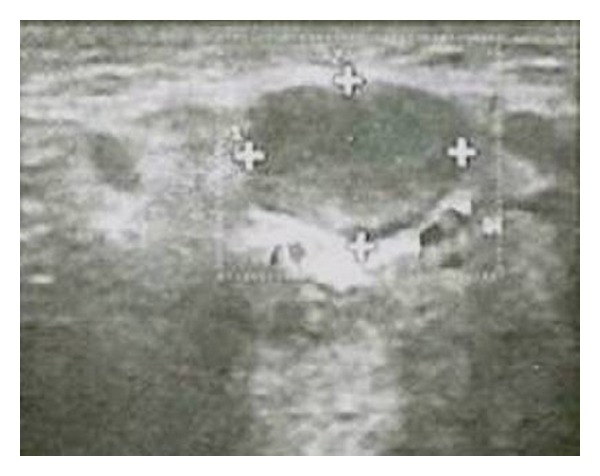
Metastatic left upper cervical lymph node measuring 2 × 1.8 cm with sharp nodal borders and absence of echogenic hilum.

**Figure 3:**
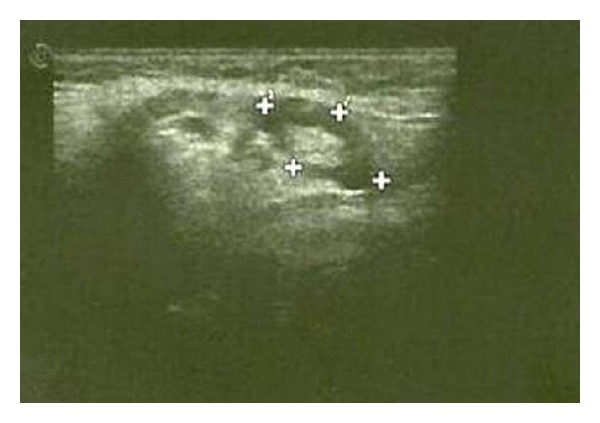
Reactive right submandibular lymph node measuring 1.62 × 0.86 cm with smooth nodal borders, hypoechoic internal structure, and presence of echogenic hilum.

The present study has shown that 100% of nodal borders of metastatic cervical lymph nodes were sharp (Figure 2) and 100% of normal and reactive cervical lymph nodes were with smooth borders (Figure 3) with highly significant *P* value (*P* = 0.0000). Sharp borders are believed to be due to the tumour infiltration and the reduced fatty deposition within the lymph nodes, which increase the acoustic impedance difference between the lymph node and the surrounding tissues. Unsharp borders are common in reactive nodes and these are due to the edema and inflammation of the surrounding soft tissue [21]. Similar results were reported by Ahuja and Ying where 94% of metastatic cervical lymph nodes had sharp nodal borders and 100% of reactive cervical lymph nodes had smooth nodal borders [5]. Esen also found similar results in their studies [22].

Echogenic hilumis the area in which the blood and lymphatic vessels drain into the lymph nodes [23]. Vassallo et al. reported that the echogenic hilus corresponds to the abundance of collecting sinuses and provides acoustic interfaces to reflect a portion of the ultrasonic wave making the hilus echogenic [24]. Yusha et al. found absence of hilar echo in 97% of metastatic cervical lymph nodes whereas 73% of nonmetastatic cervical lymph nodes showed hilar echogenicity with *P* value <0.001 [20] In our study none (100%) of metastatic cervical lymph nodes revealed absence of hilar echo (Figure 2) when compared to normal and reactive cervical lymph nodes where hilar echo was seen in all the samples (Figure 3) with highly significant *P* value (*P* = 0.0000). Similar findings were reported by Ying et al. who found echogenic hilus to be a normal sonographic feature of normal cervical lymph nodes in 96% of cases; they stated that although metastatic nodes lack this feature, hilum may be present in the early stage of involvement in which medullary sinuses have not been sufficiently disrupted to eradicate it [25]. The findings in present study can be attributed to the fact that all the malignant cases were in advanced stage.

Normal and reactive nodes were predominantly hypoechoic when compared with the adjacent muscles. Metastatic nodes were usually hyperechoic. Therefore, hyperechogenicity is a useful sign to identify metastatic nodes as stated by Ying and Ahuja [10]. Considering the internal echogenicity the present study confirmed that 60% of the metastatic cervical lymph nodes showed hyperechoic pattern of echogenicity (Figure 2) whereas normal and reactive nodes revealed 100% hypoechoic pattern of echogenicity (Figure 3) with highly significant *P* value (*P* = 0.0000). Yusha also found that interechogenic pattern was hyperechoic in 86% and 2% of metastatic and reactive cervical lymph nodes, respectively [20].

**Figure 4:**
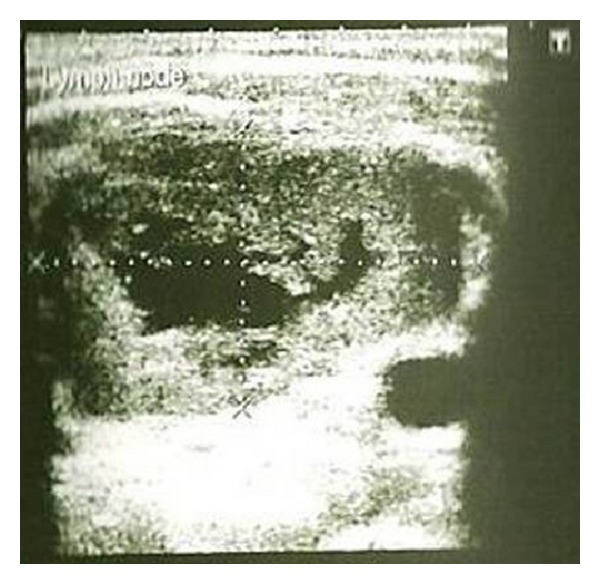
Metastatic left submandibular lymph node depicting intranodal necrosis.

**Figure 5:**
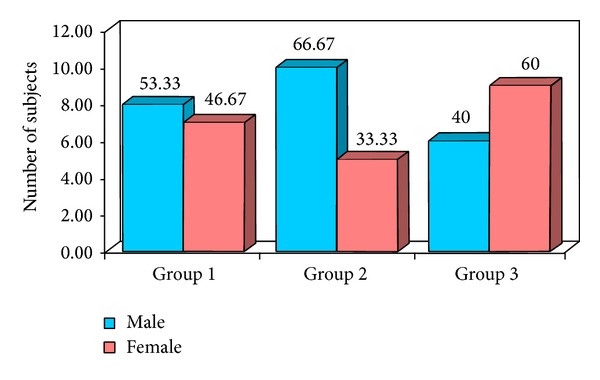
Percentage distribution of study subjects according to study groups and gender.

**Figure 6:**
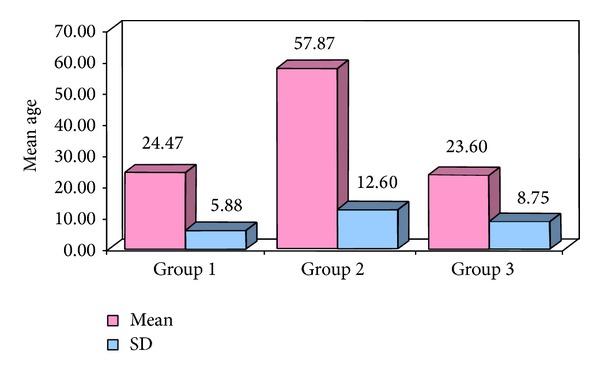
Comparison of mean and SD of age according to study groups.

**Figure 7:**
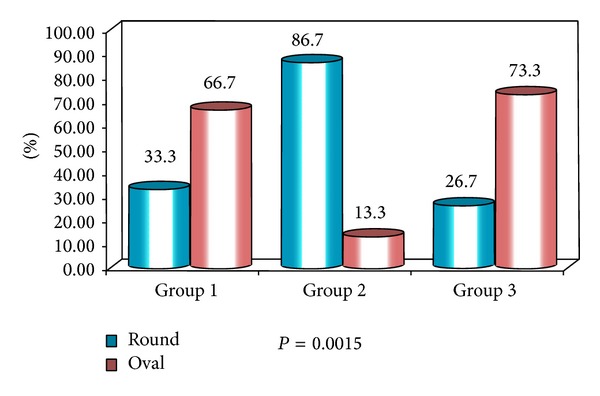
Percentage distribution of study subjects according to three groups (1, 2, and 3) and shape of lymph nodes.

**Figure 8:**
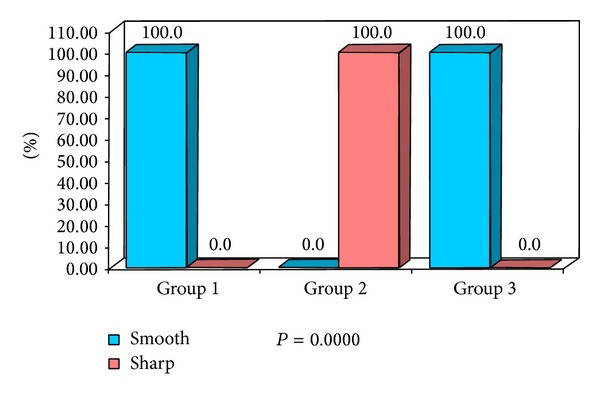
Percentage distribution of study subjects according to three groups (1, 2, and 3) and nodal borders of lymph nodes.

**Figure 9:**
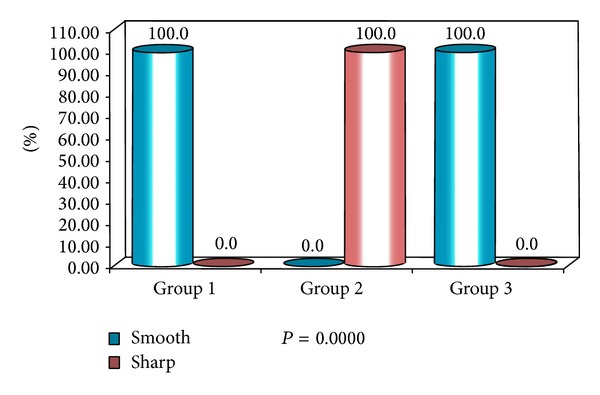
Percentage distribution of study subjects according to three groups (1, 2, and 3) and echogenic hilum of lymph nodes.

**Figure 10:**
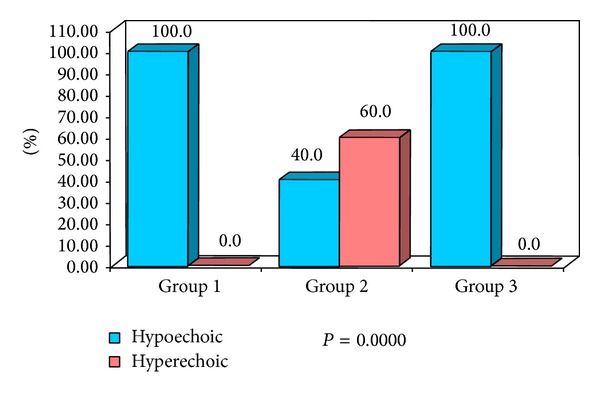
Percentage distribution of study subjects according to three groups (1, 2, and 3) and internal echogenicity of lymph nodes.

**Figure 11:**
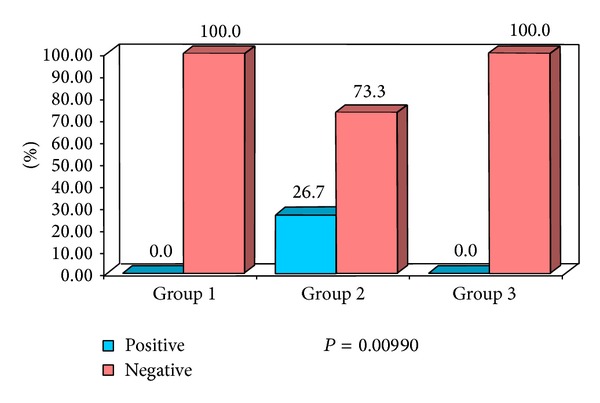
Percentage distribution of study subjects according to three groups (1, 2, and 3) with respect to intranodal necrosis of lymph nodes.

**Figure 12:**
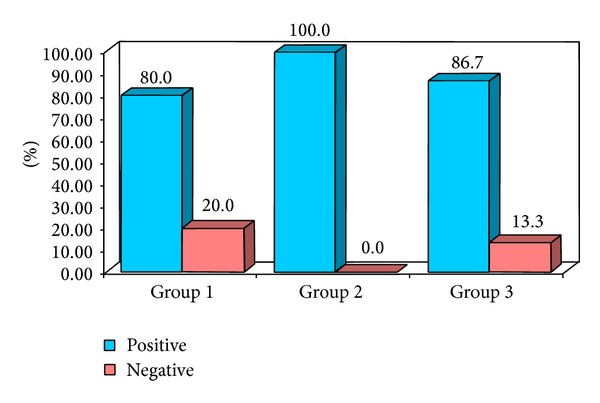
Percentage distribution of study subjects according to three groups (1, 2, and 3) with respect to U/S guided FNAC.

In the current study the intranodal necrosis was found in 26.67% of metastatic cervical lymph nodes (Figure 4) and there was no intranodal necrosis in reactive cervical lymph node with significant *P* value (*P* = 0.0099). This result was comparable to the report given by Rosário et al. [26]. Lymph nodes with intranodal necrosis were considered to be pathologic. Intranodal necrosis can be classified into coagulation necrosis and cystic necrosis, where cystic necrosis is more common than coagulation necrosis. Coagulation necrosis appears as an intranodal echogenic focus, while cystic necrosis appears as hypoechoic area within the lymph nodes. Cystic necrosis is commonly found in metastatic nodes from squamous cell carcinomas and papillary carcinoma of the thyroid [4, 27]. In the current study metastatic lymph nodes revealed cystic necrosis.

The sensitivity is the ability of a test to correctly identify those with the disease (true positive rate), whereas specificity is the ability of the test to correctly identify those without disease (true negative rate). The sensitivity and specificity of ultrasound in differentiating metastatic and nonmetastatic cervical lymph nodes, which include normal and reactive nodes, were analysed and interpreted as ultrasonography criteria such as nodal borders and echogenic hilum had high sensitivity and specificity of 100%.

## 5. Conclusion

Lymph node evaluation can be accomplished with various modalities like CT, MRI, PET, and radionuclide imaging; however these are expensive and not widely available. The present study was one such attempt to prove the efficacy of ultrasound which is nonionizing, noninvasive, cost effective, and easily available in differentiating benign and metastatic cervical group of lymph nodes. From the current study we conclude that cervical group of lymph nodes with ultrasonographic features such as round shape, absence of hilar echo, sharp nodal borders, hypoehoic internal echogenicity and presence of intranodal necrosis were highly suggestive of metastatic cervical lymph nodes; however nodal borders and echogenic hilum criteria revealed high sensitivity and specificity of 100%.

## Figures and Tables

**Table 1:** Mean and SD of size of lymph nodes of study subjects by study groups.

Group	Mean size	SD size
Group 1	0.82	0.22
Group 2	2.29	0.54
Group 3	1.24	0.20
Total	**1.45**	**0.71**

**Table 2:** Comparison of three groups (1, 2, and 3) with respect to size of lymph nodes by one way ANOVA test.

Source of variation	Degrees of freedom	Sum of squares	Mean sum of squares	*F* value	*P* value
Between groups	2	17.04	8.5178	68.1663	0.0000*
Within groups	42	5.25	0.1250		
Total	**44**	**22.28**			

*Represents highly significant *P* value.

**Table 3:** Sensitivity and specificity of ultrasonography in differentiating metastatic from benign cervical group of lymph nodes.

Summary	Size	Shape	Nodal borders	Echogenic hilum	Internal echogenicity	Intranodal necrosis
Sensitivity	83.3%	86.6%	100%	100%	60%	26.6%
Specificity	66.6%	75.4%	100%	100%	100%	100%
